# Immune dysregulation among students exposed to exam stress and its mitigation by mindfulness training: findings from an exploratory randomised trial

**DOI:** 10.1038/s41598-020-62274-7

**Published:** 2020-04-02

**Authors:** Lorinda Turner, Julieta Galante, Maris Vainre, Jan Stochl, Géraldine Dufour, Peter B. Jones

**Affiliations:** 1 Department of Medicine, University of Cambridge, Addenbrookes Hospital, Cambridge, CB2 0QQ United Kingdom; 2 Department of Psychiatry, University of Cambridge, Herchel Smith Building, Cambridge, CB2 0SZ United Kingdom; 3 National Institute for Health Research Applied Research Collaboration East of England, Cambridge, United Kingdom; 4 Praxis Centre for Policy Studies, Tartu mnt 50, Tallinn, 10115 Estonia; 5 Medical Research Council Cognition and Brain Sciences Unit, University of Cambridge, 15 Chaucer Road, Cambridge, CB2 7EF United Kingdom; 6 University Counselling Service, University of Cambridge, 2-3 Bene’t Place, Lensfield Road, Cambridge, CB2 1EL United Kingdom; 7 British Association for Counselling & Psychotherapy: Universities and Colleges Division, Cambridge, United Kingdom; 8 Department of Kinanthropology, Charles University, Prague, Czech Republic

**Keywords:** Immunology, Psychology

## Abstract

Psychological distress persisting for weeks or more promotes pro-inflammatory immune dysregulation, a risk factor for a range of chronic diseases. We have recently shown that mindfulness training reduces distress among university students. Here we present an exploratory trial to study immune dysregulation in a cohort of students who were exposed to progressively greater stress as the exam period approached, and to explore whether mindfulness training mitigated this dysregulation. Healthy University of Cambridge students were randomised to join an 8-week mindfulness course (N = 27), or to mental health support as usual (N = 27). Psychological distress, immune cell proportions, cytokines, CRP and serum cortisol were measured at baseline and during the exam period. Increased distress was associated with statistically significant increases in the proportion of B cells, regardless of trial arm (*p = 0.027). There were no other associations between any of the measured parameters, distress or mindfulness. Our finding that the proportion of B cells increases with psychological distress supports the findings of other studies. However, we found no evidence that mindfulness training is able to buffer the effects of psychological distress on healthy participants’ immune system. In order to detect these effects, should they exist, larger randomised trials will be required.

## Introduction

The human mind, brain and immune systems have much in common. They are complex, self-organising economies that recognise self from non-self, interact with the environment and possess ability to store information. The immune system alone is a highly distributed network of effectors, regulators and signals, which depend on dynamic balances to determine the best configuration for each environmental challenge.

Prolonged systemic inflammation leads to increased risk of chronic diseases such as cancer, cardiovascular disease, diabetes, dementia and depression^1^, the most important global causes of morbidity and mortality. Thus, interventions that restore or maintain normal immune function in healthy and at risk populations are useful in preventing a range of mental and physical disorders^2,3^. Even small improvements at a population level could lead to large reductions in morbidity and mortality^4^.

The immune, endocrine and nervous systems are known to interact with one another to provide a co-ordinated response to external stimuli, whether it be a physical insult such as wounding or an emotional stressor such as perceived stress (reviewed in Glaser *et al*.)^5^. Acute stress promotes a pro-inflammatory state along with the “fight or flight” response; if stress persists for weeks or more (chronic stress) this pro-inflammatory state fails to resolve adequately, resulting in damage^6^.

The effects of stress on immune function are thought to be mediated in part by the steroid hormone cortisol which may increase during psychological distress. Modulation of immune function by cortisol and other hormones could be mediated by direct interaction with immune cells or sympathetic nervous system fibres^7^, or indirectly by dysregulating the production of cytokines^5,8,9^. A popular model that could explain the pattern of chronic disease empirically linked with chronic stress hypothesizes that cortisol and other stress factors suppress cellular (Th1) adaptive immunity, while humoral (Th2) adaptive immunity is overstimulated^10^. Indeed, several studies have demonstrated shifts in favour of Th2 cytokine imbalance in response to stress^11,12^.

A comprehensive meta-analysis of non-clinical examination stress studies found support for this model via a decrease in interferon-γ (IFN- γ) and increases in interleukin-6 (IL-6) and IL-10^8^. However, pro- and anti- inflammatory cytokines are produced by varied immune cell subsets and it is difficult to interpret changes in peripheral blood protein levels without an understanding of the cellular response to stress, both within and beyond the T cell compartment. Furthermore, chronic stress leads to glucocorticoid resistance and desensitisation leading to the failure of cortisol to suppress inflammation independently of its blood level^13^.

A meta-analysis performed by Segerstrom and Miller found that NK cell activity and proportions of certain lymphocyte subsets were increased, but lymphocyte proliferation overall decreased following public speaking, an acute stressor^8^. Given that most immune cell subsets have receptors that recognise and can respond to neurotransmitters and hormones associated with stress, it is plausible that these changes in immune cell numbers, proliferative capacity and function could be in response to stress hormones and sympathetic innervation of lymphoid organs^5^. Over the last several decades many advances have been made towards understanding how the immune, endocrine and nervous systems interact and may become dysregulated during times of psychological distress. The studies mentioned above have contributed to our knowledge, but we still lack precise mechanistic understanding of how specific components of the immune system contribute to psychological distress and vice versa.

Healthy, young but stressed individuals may greatly benefit from learning lifelong skills to manage their stress and therefore prevent immune dysregulation which, if maintained throughout life, could lead to chronic disease. Teaching those skills to students could not only help to support their wellbeing and mental health when they are at university but potentially for the rest of their lives. At a time when increasing number of young people are pursuing university education, this could have huge health improvement applications.

Mindfulness meditation is a way of training the attention and its regulation for the purpose of promoting mental health. Within mindfulness-based programmes, mindfulness practice has been frequently defined as the awareness of paying attention to the present moment internally and externally, with an attitude of non-judgemental kindness and curiosity^14^. Mindfulness-based programmes may be described as trans-diagnostic interventions, associated with improvements on various health outcomes within both clinical and non-clinical settings^15^. There is evidence for the effectiveness of mindfulness-based programmes to prevent psychological distress^16^, and help to mitigate the negative symptoms of common mental disorders such as depression and anxiety^17^. Several theoretical models of mindfulness have been proposed depicting specific psychological mechanisms of action underlying these effects. The most commonly-cited mechanisms include decentering, the regulation of attention and/or self, self-compassion, and acceptance^18–24^.

Heightened attention and/or self-regulation emerging from mindfulness practice may reduce the extent to which potential social stressors are cognitively evaluated as threats ^15,25,26^. This may in turn reduce sympathetic activation and consequently pro-inflammatory immune response. A small but growing number of studies are assessing whether mindfulness training can influence the immune system. A recent meta-analysis combining randomised trials of meditation techniques in clinical and non-clinical samples found reductions in blood cortisol, C-reactive protein (CRP) and tumour necrosis factor-α (TNF-α) but no change in IL-6 compared with active controls^27^. The only consistent findings of a systematic review of randomised trials looking at mindfulness training in different populations were reductions in CRP, and increases in CD4^+^ T cell count among HIV-diagnosed individuals^28^. Null findings or a lack of replicated effects were found for antibodies, interleukins IL-1, IL-6, IL-8 and IL-10, IFN-γ, TNF-α, and various measures of immune cell proportion and functionality. Studies performed by Linda Witek-Janusek and colleagues demonstrated that women recently diagnosed with breast cancer had reductions in peripheral blood NK cell activity and IFN-g production, accompanied by increased IL-4, IL-6 and IL-10^29,30^. Interestingly, following mindfulness-based stress reduction cortisol levels were reduced and normal NK cell function re-established.

Thus, the effects of mindfulness training programmes on immunological biomarkers are less clear within non-clinical than clinical populations^31^. Few basic immunological outcomes have been tested in randomised trials of community samples^32–40^. We aimed to conduct a well-controlled pre-post exploration of how mindfulness buffers the effects of stress on the normal immune system in a typically healthy but psychologically distressed population of university students^41^.

Among undergraduates at the University of Cambridge stress peaks during the exam term - two months of revision and examinations which determine the outcome of their entire academic year; grades can play a crucial role in defining their career paths. In 2015 the University of Cambridge funded the Mindful Student Study: a randomised, waiting-list controlled trial of an 8-week manualised mindfulness program adapted to students^42^. In this trial we showed that mindfulness training reduces psychological distress among university students, particularly during the exam revision period, improving resilience to stress. We conducted a small exploratory trial alongside the Mindful Student Study to study immune dysregulation and inflammation stemming from this cohort of students who were exposed to progressively greater stress as the exam period approached, and to explore whether mindfulness training could help mitigate this dysregulation.

## Results

**Figure 1:**
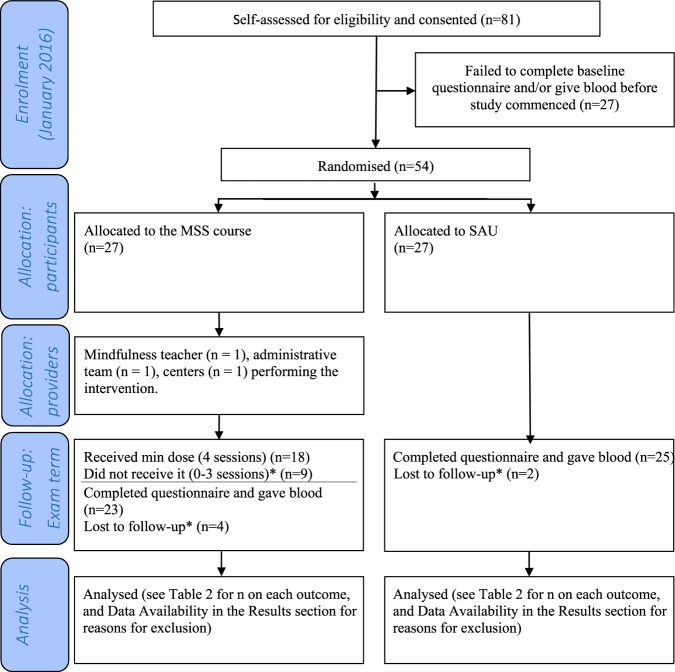
CONSORT 2010 flow diagram. *Reasons explained in text. Abbreviations: min = minimum, MSS = Mindfulness Skills for Students; SAU = support as usual.

Figure 1 describes the flow of participants. The trial ran and ended as planned. Participants randomised to the intervention attended a median of seven sessions (inter-quartile range 2–7) including one person who attended no sessions at all, and six students who attended all the sessions. Eighteen participants (67% of those randomised to the intervention) attended at least half of the mindfulness course sessions, our pre-specified “minimum dose”^42^ also used in previous studies^43^, and shown to produce meaningful change^44^. Three participants provided reasons for abandoning their mindfulness course; these were schedule conflicts or being too busy. Six participants (11%) failed to provide outcome data. No participants had adverse reactions related to self-harm, suicidality, or harm to others^16^.

Table 1 shows participants’ characteristics, and Table 2 shows outcome values at all time points. In small trials randomisation can still produce imbalanced groups by chance; in our sample control group participants had a larger body mass index, a plausible confounder, therefore we have controlled for it in sensitivity analyses. However, none of the baseline random imbalances were “statistically significant”.

**Table 1:** Participants’ characteristics.

		Control		Intervention		Total	
		N	%	N	%	N	%
Gender	Females	20	74.07%	18	66.67%	38	70.37%
Age	17–21	11	40.74%	13	48.15%	24	44.44%
	22–30	12	44.44%	12	44.44%	24	44.44%
	31+	4	14.81%	2	7.41%	6	11.11%
Nationality	UK or European Union	16	59.26%	20	74.07%	36	66.67%
Ethnicity	Asian	6	23.08%	5	20.00%	11	21.57%
	Black	1	3.85%	0	0.00%	1	1.96%
	Mixed	2	7.69%	0	0.00%	2	3.92%
	Other	0	0.00%	3	12.00%	3	5.88%
	White	17	65.38%	17	68.00%	34	66.67%
Disability	Yes	1	3.70%	4	14.81%	5	9.26%
Degree	Undergraduate	12	44.44%	12	44.44%	24	44.44%
	Masters	1	3.70%	3	11.11%	4	7.41%
	MPhil	5	18.52%	5	18.52%	10	18.52%
	PhD	9	33.33%	7	25.93%	16	29.63%
Last Year	Yes	14	51.85%	13	48.15%	27	50.00%
BMI	Underweight	0	0%	2	9%	2	4%
	Healthy weight	14	56%	15	65%	29	60%
	Overweight	7	28%	5	22%	12	25%
	Obese	4	16%	1	4%	5	10%

**Table 2:** Outcome values for all time points and groups, and statistical tests for pre-post changes on the total sample.

		Baseline					Exam term				
		N	Mean	SD	Median	Range	N	Mean	SD	Median	Range
CORE-OM	MSS	26	0.93	0.49	0.87	0.18 to 2.06	25	1.01	0.61	0.85	0.09 to 2.29
	Control	27	0.9	0.54	0.76	0.03 to 0.2.32	27	1.05	0.52	1	0.06 to 2.06
	Total	53	0.91	0.51	0.82	0.03 to 2.32	52	1.03	0.56	0.96	0.06 to 2.29
Cortisol	MSS	26	313.65	123.48	325	109 to 518	22	324.55	201.82	300	121 to 992
	Control	27	353.96	199.15	303	118 to 896	25	289.32	168.97	205	119 to 781
	Total	53	334.19	166.06	316	109 to 896	47	305.81	183.87	228	119 to 992
IL-8	MSS	26	8.43	3.29	7.9	3.9 to 17.6	22	9.35	3.86	8.15	5.1 to 21.4
	Control	27	7.24	2.54	6.6	3 to 14.4	25	8.85	3.67	8.2	4.3 to 21.6
	Total*	53	7.82	2.97	7	3 to 17.6	47	9.08	3.72	8.2	4.3 to 21.6
TNF-α	MSS	26	1.78	0.43	1.75	1 to 2.7	22	2.12	0.7	1.85	1.4 to 4.2
	Control	27	1.74	0.39	1.7	1.2 to 3.1	25	1.97	0.43	1.8	1.5 to 3.5
	Total**	53	1.76	0.4	1.7	1 to 3.1	47	2.04	0.57	1.8	1.4 to 4.2
CRP	MSS	26	1.84	2.16	1.3	0.5 to 11.7	22	1.32	1.23	0.75	0.5 to 4.4
	Control	27	2.15	1.89	1.4	0.9 to 8.8	25	1.78	2.24	1	0.5 to 9.4
	Total^*^	53	2	2.01	1.4	0.5 to 11.7	47	1.57	1.83	1	0.5 to 9.4
% CD4^+^ T Lymphocytes	MSS	24	0.62	0.1	0.64	0.33 to 0.75	20	0.6	0.09	0.62	0.45 to 0.73
	Control	26	0.65	0.11	0.66	0.39 to 0.82	25	0.59	0.1	0.59	0.33 to 0.76
	Total^*^	50	0.63	0.1	0.65	0.33 to 0.82	45	0.6	0.1	0.62	0.33 to 0.76
% CD8^+^ T Lymphocytes	MSS	24	0.23	0.1	0.21	0.14 to 0.58	20	0.28	0.08	0.26	0.18 to 0.47
	Control	26	0.19	0.08	0.17	0.09 to 0.5	25	0.29	0.08	0.29	0.15 to 0.5
	Total^***^	50	0.21	0.09	0.18	0.09 to 0.58	45	0.29	0.08	0.28	0.15 to 0.5
% CD19^+^ B Lymphocytes	MSS	24	0.15	0.09	0.14	0.03 to 0.37	19	0.22	0.08	0.21	0.05 to 0.34
	Control	26	0.16	0.07	0.16	0.05 to 0.29	25	0.25	0.09	0.23	0.08 to 0.4
	Total^***^	50	0.16	0.08	0.16	0.03 to 0.37	44	0.23	0.08	0.23	0.05 to 0.4
% CD14^+^ Monocytes	MSS	24	0.35	0.12	0.33	0.12 to 0.6	18	0.42	0.1	0.4	0.22 to 0.61
	Control	26	0.38	0.1	0.37	0.16 to 0.58	24	0.45	0.12	0.47	0.22 to 0.61
	Total**	50	0.37	0.11	0.36	0.12 to 0.6	42	0.43	0.11	0.42	0.22 to 0.66
% NK cells	MSS	24	0.25	0.13	0.21	0.09 to 0.63	18	0.29	0.13	0.29	0.11 to 0.49
	Control	26	0.18	0.09	0.15	0.06 to 0.44	24	0.24	0.11	0.22	0.08 to 0.54
	Total**	50	0.21	0.11	0.2	0.06 to s0.63	42	0.26	0.12	0.23	0.08 to 0.54

*p < 0.05; **p < 0.01; ***p < 0.001. All p values are adjusted for multiple comparisons. Abbreviations: MSS: Mindfulness Skills for Students course; NK: natural killer.

Data from two participants did not pass quality controls and were removed from further analysis. Only four blood biomarkers had levels that were above the limit of quantification for the majority of participants: cortisol, CRP, IL-8, and TNF-α. Cortisol is a hormone that is thought to mediate the effects of chronic stress on the immune system through complex interplays; CRP, IL-8, and TNF-α are pro-inflammatory proteins secreted by cells during the course of a normal pro-inflammatory immune response^8^.

We performed a PLS analysis to determine the combinations of cell classes (components) which best explained variation in distress levels measured during the exam period. Predictor variables were the 154 cell classes and the response variable was the CORE-OM score. Permutation testing of the model was not significant (p = 0.738), precluding inference about the contribution of specific cell classes to the model (Supplementary figure S2). We therefore manually chose cell classes based on prior evidence within the scientific literature.

### Immune responses to stress

As a preliminary check, we performed a full-sample pre-post comparison of outcome values to assess the effects of the stressful exam revision period on the immune system. Several factors related to inflammation were significantly increased the second time participants were sampled, which was during the exam period: IL-8 (*p < 0.05), TNF-α (**p < 0.01), CD8^+^ T cells (***p > 0.001), B cells (***p < 0.001), monocytes (**p < 0.01), and NK cells (**p < 0.01). Conversely, CRP (*p < 0.05), CD4+ T cells (*p < 0.05) were significantly decreased (after correction for multiple comparison testing) when sampled during the exam period (Table 2).

**Figure 2:**
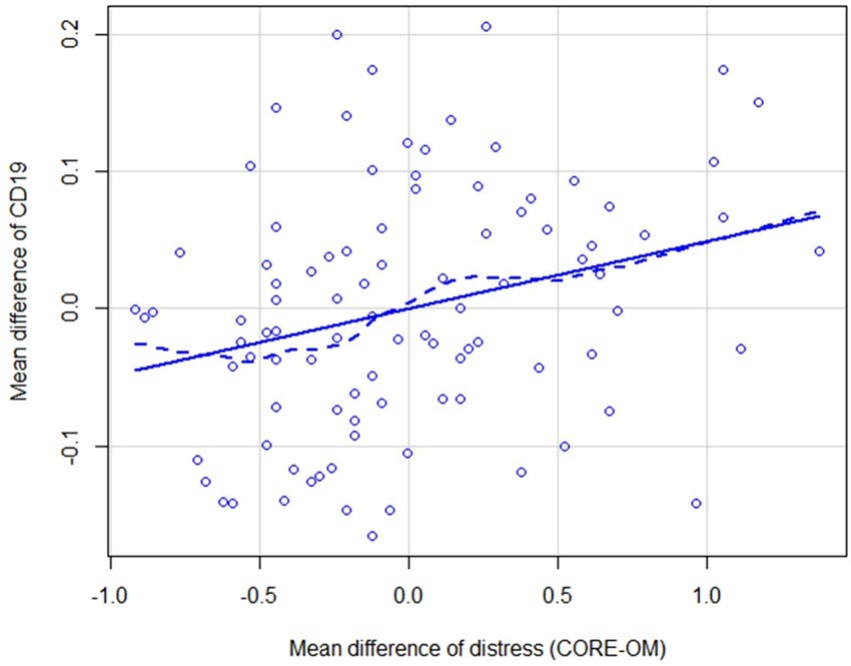
Relationship between mean differences of CORE-OM and mean differences of CD19. To obtain the mean differences, the distances of each CORE and CD19 data point from the mean CORE and CD19 at their respective time points were calculated.

Table 3 summarises the analyses assessing how changes in perceived distress levels correlated with changes in blood biomarkers and cell proportions. The proportion of B lymphocytes significantly increased by 0.05 with every one-point increase in the distress scale (p adjusted for multiple comparisons = 0.027); as a sensitivity analysis we adjusted this model for body mass index and the result was maintained. The effect is visually described in Fig. 2 (similar figures for all outcomes are shown in supplementary figures S3 and S4). There were no other significant relationships between biomarkers and distress levels.

**Table 3:** Summary of random intercept models looking at fixed effects for change in distress.

Outcome (unit)	Regression coefficient^a^	Standard error	P-value^b^
Cortisol (nmol.L)	−45.67	34.69	0.95
CRP (mg.L)	0.52	0.33	0.77
IL-8 (pg.ml)	0.48	0.63	1
TNFa (pg.ml)	−0.07	0.10	1
% CD4+ T Lymphocytes	0.04	0.02	0.40
% CD8+ T Lymphocytes	−0.03	0.02	0.77
% CD19+ B Lymphocytes	0.05	0.02	**0.027**
% CD14+ Monocytes	0.03	0.02	0.95
% NK cells	−0.002	0.02	1

^a^Adjusted mean difference. ^b^All p values are adjusted for multiple comparisons. Abbreviations: NK: natural killer.

### Effects of mindfulness training

Table 4 summarises the analyses assessing the effects of mindfulness training on blood biomarkers and immune cells. There were no significant effects on any of the parameters (unadjusted p = 0.03 for the effect on NK cells). Results were similar in the per-protocol analyses.

**Table 4:** Summary of random intercept models looking at fixed effects for trial arm (ITT analysis).

Outcome (unit)	Regression coefficient^a^	Standard error	P-value^b^
Cortisol (nmol.L)	−2.34	40.92	1
CRP (mg.L)	−0.40	0.49	1
IL-8 (pg.ml)	0.94	0.84	1
TNFa (pg.ml)	0.08	0.11	1
%CD4+ T Lymphocytes	−0.01	0.03	1
%CD8+ T Lymphocytes	0.02	0.02	1
%CD19+ B Lymphocytes	−0.02	0.02	1
%CD14+ Monocytes	−0.03	0.03	1
%NK cells	0.07	0.03	0.27

^a^Adjusted mean difference. ^b^All p values are adjusted for multiple comparisons. Abbreviations: ITT: intention-to-treat; NK: natural killer.

## Discussion

In our opportunistic study within a larger trial, healthy university students were exposed to an objectively stressful insult – exam revision – persisting for several weeks, but their subjective experience of psychological distress varied. We set out to test whether participants’ changes in subjective experience were linked to stress-related pro-inflammatory immune change, and whether mindfulness training would buffer these effects.

Regardless of whether the students underwent mindfulness training or not, several immune parameters were significantly increased across the whole cohort during the exam period when compared with the baseline measurements. A potential confounding factor lies in the season of sampling; all baseline measurements were collected in January (winter) whereas the exam period measurements were collected in June (spring). Relatively little is known regarding seasonal changes in immune cell populations. One study found that seasonal gene expression changes in peripheral blood mononuclear cells (PBMCs) strongly correlated with the expression levels of genes known to mark different immune cell types^45^, indirectly suggesting that the cellular composition of peripheral blood changes with the season. A second study found that complete blood count was subject to seasonal variation, however this study did not address changes to specific immune cell subsets such as B cells and NK cells^46^. It is possible that these changes across the entire cohort in our study may represent, in part, seasonal effects. Regardless, for all but the CD19+ B cells, these effects were not related to measures of perceived psychological distress.

A distinctive feature of B cells is that they produce antibodies; an increase in their proportion may indicate elevated immune activity that may in turn increase chronic tissue inflammation^47,48^. Our finding that the proportion of B cells increases with increased psychological distress (Table 3 and Fig. 2) is in line with previous research on exam stress in university samples. In a recent cohort study, students B cells increased significantly with exam stress compared to community controls while awakening cortisol response flattened^49^. B cells however decreased when controlling for awakening cortisol response. It is possible that this could be due to negation of the mediating influence of decreased cortisol on upregulation of B cell production. Further supporting our findings, Maes *et al*. assessed students the day before examinations and found several changes, among them an increase in the absolute numbers of B cells^50^. Very little is known about the precise role of B cells in psychological distress. Only a handful of studies have demonstrated alterations in the proportion or activity of B cells as a result of psychological distress, and to the best of our knowledge no mechanism has yet been proposed.

Regarding our null findings in other parameters, Segerstrom *et al*. also did not find evidence of TNF-α being effected by exam stress in a meta-analysis of a hundred healthy individuals^8^. In other studies, academic stress did not elevate cortisol levels^51^, and there were no correlations between plasma cortisol levels and type-1/type-2 cytokine production^52^. The effect size coefficients suggest there could be an inverse relationship between cortisol and distress. This was not statistically significant, but this could be due to lack of statistical power, as suggested by the large dispersion measures.

In healthy individuals immune parameters are relatively stable within a range^53^. Distress may need to surpass a threshold for the immune system to be triggered. The immune system is remarkably flexible and capable of substantial change without compromising an otherwise healthy host^8^. A comprehensive but somewhat dated meta-analysis of examination stress studies did not find evidence for this type of stress, either objectively or subjectively experienced, markedly affecting the number or percentage of cells in peripheral blood^8^. An alternative explanation for these null findings is some immunological changes related to stress may be relatively restricted to the brain and meningeal immune compartments, and not reflected (or less easily detected) in peripheral blood cells. The immunophenotyping used in our study is far more comprehensive than any previously published studies to our knowledge, but we still detected few meaningful cell percentage changes. Cell changes among clinical populations may be easier to detect, e.g. asthmatic students^54^.

Our controlled pre-post analysis represents significant progress compared to evidence coming from cross-sectional samples. We applied multiple comparison corrections and leveraged multivariate techniques to ensure a robust analysis of the high-dimensional data generated by this study. However, given the small sample size, we were unable to adjust findings for plausible confounders such as body mass index (for which we only have controlled one sensitivity analysis), age or gender. There is a need for adjusted replication in larger samples.

Loss to follow up (11%) may have biased our sample in ways that are hard to predict, although such figures are to be expected in trials testing non-clinical behavioural interventions^55^. In addition, many cytokine analyses were limited by sample levels being below reliable quantification ranges in our analyses. Indeed, cytokine levels are generally low in otherwise healthy individuals, so quantification methods may need higher sensitivity to measure these reliably.

With respect to the effects of mindfulness training, we know from the Mindful Student Study, conducted on a larger sample of 616 students, that it significantly reduces self-reported psychological distress with a moderate effect size^16^. The present study could not clearly detect how this reduction in distress generated by mindfulness training translates into changes in immune parameters related to inflammation. However, based on our data we cannot discard a biological linkage between mindfulness and distress. There was an initial indication that the proportion of NK cells may increase with mindfulness training, but statistical significance did not survive multiple comparison correction. However, a previous non-randomised trial suggested that mindfulness training increases NK cell activity among women recently diagnosed with breast cancer^30^, and lower levels of NK activity were detected among healthy individuals who were more distressed than their peers^8,56^.

NK cells play a major role in the rejection of both tumour and virally infected cells; higher levels may indicate a stronger defence against these threats but the functional implications for chronic tissue inflammation are still unclear^57,58^. In any case, despite the methodological strengths of our randomised design (mainly the minimization of confounding effects), a larger trial will be required to clarify the effect of mindfulness training on the proportion of NK cells. Alternatively, this could be clarified by increasing the number of repeated measures within a shorter period and more proximal to the stressor. It is plausible that whilst there is only a marginal change in the proportion of NK cells, there may be alterations to their cytolytic activity or gene expression profiles which were not investigated in the current study.

More generally, Black *et al*. did not find evidence that mindfulness training affects IL-8, TNF-α or immune cell counts^28^. Other randomised trials of community samples also failed to find effects on CRP^35,59^ and cell counts^60^, although mindfulness meditation significantly influenced TNF-α levels in healthy individuals when individual studies were combined in a meta-analysis. Trials on clinically anxious or depressed populations found significant effects on TNF-α with samples of 60–70 participants^61,62^; however a recent actively controlled trial with HIV patients failed to find any effects^63,27^.

In line with our trial, mindfulness training has repeatedly failed to show evidence of reducing salivary cortisol reactivity to acute stress in randomised trials of community samples^32,36,38,59^. In a trial conducted in Germany with 313 volunteers from the community, after 3-month mindfulness-based attention training, self-reported stress reactivity to the Trier Social Stress Test (TSST) was reduced but salivary cortisol stress response was increased (non-significantly) relative to no training^40^. Three-day mindfulness meditation training in a trial assessing university students in the United States also reduced self-reported stress reactivity but significantly *increased* salivary cortisol reactivity, relative to the cognitive training controls^39^. The latter result might be explained by a subconscious increase in distress during the early stages of mastering emotion regulation mindfulness skills, when decentering and interoceptive awareness have not been well developed yet. However, this post-hoc explanation would need to be tested in a purposively designed prospective study.

This small exploratory study contributed to the existing literature by strengthening the evidence on some promising directions regarding stress, mindfulness and immune dysregulation. Our finding that the proportion of B cells increases with increased psychological distress is robust and confirms previous research, but needs well-adjusted replication. If mindfulness training is to buffer the effects of psychological distress on the immune system of healthy populations under stressful situations, randomised trials with larger samples, more measurement time points, and perhaps more intense training, will be required to detect it. A larger sample size would additionally allow the researcher to investigate the effects of the ‘dose’ of mindfulness-based interventions on immune cell parameters. In order to avoid multiple testing, future trials could focus their efforts on NK cells and B cells which our study has shown may be promising directions.

## Methods

The Cambridge Psychology Research Ethics Committee approved this study on 16/12/2015 (PRE.2015.098). Informed consent was obtained from all participants, and volunteer participants followed all the steps of the Mindful Student Study randomised controlled trial in accordance with guidelines and regulations^16,42^. The trial was registered with the Australia and New Zealand Clinical Trials Registry on 30/10/2015, number ACTRN12615001160527.

In January 2016 University of Cambridge students without severe mental illness or crisis self-enrolled online and were randomly allocated (1:1), automatically via remote survey software using computer-generated random numbers, to join an 8-week mindfulness course adapted for university students plus mental health support as usual (intervention group), or to mental health support as usual alone (control group). Mental health support as usual consisted of the opportunity to access centralised support at the University of Cambridge Counselling Service (UCS) in addition to pastoral support at university college level (Cambridge is a collegiate University comprising 31 Colleges which admit, accommodate, tutor and teach students), and public health services external to the University. Those allocated to the control group were offered a mindfulness course one year later, providing they were still students at the University.

The primary outcome was self-reported psychological distress during the main examination period in the University academic calendar (May-June 2016). Please see trial publications for more details on procedures such as randomisation, questionnaire data collection, adverse event monitoring, et cetera^16,42^.

The inclusion criteria for participation in the sub-study reported here were: (a) Mindful Student Study participant recruited in January 2016; (b) having an exam, viva, 1st year PhD report, or dissertation deadline between 15 May & 15 July 2016 (self-reported). The exclusion criteria, all self-reported, were: (a) personal or family history of autoimmune disorders, severe allergy or asthma; (b) being on regular steroid medication; (c) illegal drug or alcohol dependence (addiction); (d) having meditated for more than 10 hours in total in the past or having done a mindfulness 8-week course. Sample size for this sub-study was determined based on resource availability given the costs of immunophenotyping. Hence the pilot nature of this sub-study.

The intervention, called Mindfulness Skills for Students, consisted of a secular, face-to-face, group-based skills training programme based on the course book ‘Mindfulness: A Practical Guide to Finding Peace in a Frantic World’^64^, and adapted for university students. Adaptations were focused on permeating every session with elements of flexibility, self-discovery, self-compassion and empowerment, aimed at generating a natural transfer of skills developed in meditation to study, decision-making and relationships. This course aimed to optimise wellbeing and resilience across a range of students and was not specifically developed for those students in the clinical range. An experienced and certified mindfulness teacher delivered seven, parallel courses between late January and March 2016 (i.e., all the participants randomised to the intervention did the course at the same time); each course accommodated up to 30 students and comprised eight, weekly sessions lasting 75–90 minutes.

### Measures and procedures

Psychological distress was measured using the Clinical Outcomes in Routine Evaluation Outcome Measure (CORE-OM), a 34-item generic questionnaire which has been widely used with UK university students^65^. A higher score indicates increased distress. The total mean score (range 0–4) is obtained by dividing the total score by the number of completed items (as long as no more than three items have been missed)^66^. CORE-OM has good convergent validity, internal and test-retest reliability and sensitivity to change^67^.

Participants in the present study consented to donate two blood samples on top of completing the Mindful Student Study CORE-OM questionnaire. One sample was taken before participants were randomised (baseline measurement) and the second sample approximately 3–4 months after randomisation (exam period measurement). At both time points, the CORE-OM questionnaires were collected within a week of blood sampling.

We used state-of-the-art immunophenotyping to examine the proportions of over 150 different key peripheral immune cell subsets to explore the activation patterns that emerge from exposure to chronic stress, and how mindfulness training modifies these patterns. Methods, staining procedure, antibody combinations, instrument configuration and gating strategies are described in the supplementary information. Immunophenotyping by multi-colour flow cytometry is the most powerful technology available for the analysis of population dynamics, cellular phenotype and function in the immune system^68^. It systematically evaluates the unique repertoires of cell subsets and activation markers. In addition, we measured key molecules associated with inflammatory processes: cortisol, pro- and anti-inflammatory cytokines (IL-2, IL-4, IL-6, IL-8, IL-10, IFN-γ, TNF-α, IL-1b, IL-12p70, IL-13), Epstein Barr virus and cytomegalovirus antibodies, and CRP.

All participants provided two samples of up to 22.5 mL venous blood. Blood were drawn from a butterfly placed in the forearm while participants were lying quietly on an examination couch in a clinical research facility at Addenbrookes Hospital (Cambridge, UK) with medical supervision. Their height and weight were measured using standard scales. Participants were also asked a few standard medical questions by a member of the research team (e.g., “Do you feel sick today?”) to collect information on possible recent/ongoing inflammatory processes that could explain any abnormal results in their blood on that day. As a token of appreciation, £20 were offered to each participant per sampling session in the form of Amazon vouchers.

Samples for each individual were given a unique, anonymised code and immediately subdivided for the different analyses. The researchers processing the samples were unaware of the identities of the individuals who gave the samples and had no access (blind) to questionnaire and trial arm data.

Cytokines were analysed in duplicate using the MesoScale Discovery multiplexed panel as per manufacturer’s instructions. Only cortisol, CRP, IL-8, and TNF-α were reliably within the range of detection of the assay. Immunophenotyping was performed as described in the supplementary information (SI) using the antibody panel described in SI Table 1 and SI Table 2. Leukocyte populations were expressed as relative proportions of the parent population.

### Analysis

This was an exploratory study to generate biological hypotheses for further testing. However, previous research suggests the following pre-specified hypotheses:

- The increase in perceived distress as a result of exams approaching generates a dysregulated pro-inflammatory state, characterised by increased proportions of monocytes and activated lymphocytes, accompanied by increases in pro-inflammatory humoral immune factors^6,69^. To test this hypothesis, we used random intercept models including each cell-type/humoral marker and psychological distress. The small sample size precluded further adjusting.
- Individuals trained in mindfulness will show less pro-inflammatory dysregulation^35,62^. To test this hypothesis, we used random intercept models including each cell-type/humoral and arm data. The analyses followed the intention-to-treat principle, but per-protocol analyses were also conducted as sensitivity analyses to explore the impact of adherence to the intervention. In the per-protocol analyses only those intervention participants who received our pre-specified “minimum dose” (i.e. attended at least half of the mindfulness course sessions) were included.

The number of cell classes returned by this type of immunophenotyping is very large, therefore in order to pre-select the most promising cell classes and thus reduce the number of predictors we used two strategies: data driven, and theory driven. For the data driven strategy we conducted a partial least squares (PLS) analysis using all of the 154 cell classes that were enumerated by the immunophenotyping analysis. PLS is a multivariate technique used to identify associations between a response variable, in this case psychological distress, and a set of predictors, here the relative immune cell counts. Permutation test and bootstrap were used to assess accuracy and confidence intervals, respectively (method described in full in Fernandez-Egea, E. *et al*.^70^). For the theory-driven strategy we looked at those cell classes implicated in previous distress and/or mindfulness studies: CD4^+^ and CD8^+^ T cells^28^, B cells^49^, NK cells^30^ and CD14 monocytes^50^.

To further avoid spurious results due to multiple testing, we corrected the p values using the Holm adjustment^71^. Mixed linear models were used for the analyses, which make efficient use of all available data in small samples^72^. Analyses were conducted at an alpha level of p = 0.05 (two-sided) using ‘R‘ 3.4.4. The current sample was independent of that included in the other Mindful Student Study analyses.

## Supplementary information

Supplementary information.

## Data Availability

Anonymised data available on request.
