# Supplementary material for: Immune dysregulation among students exposed to exam stress and its mitigation by mindfulness training: findings from an exploratory randomised trial
Source: Sci Rep. 2020 Apr 2;10:5812. doi: 10.1038/s41598-020-62274-7 (PMC7118166; doi:10.1038/s41598-020-62274-7)
Supplement: Supplementary file 1 — Supplementary information. [file 41598_2020_62274_MOESM1_ESM.docx]

Immune dysregulation among students exposed to exam stress and its mitigation by mindfulness training: findings from an exploratory randomised trial

***Supplementary Methods***

Lorinda Turner^#^ (1), Julieta Galante^#^*(2), Maris Vainre (3,4), Jan Stochl (2), Géraldine Dufour (5), Peter B Jones (2)

# contributed equally to this work

* Corresponding author – correspondence to mjg231@cam.ac.uk

1. Department of Medicine, University of Cambridge, Addenbrookes Hospital, CB2 0QQ, United Kingdom.
2. Department of Psychiatry, University of Cambridge, Herchel Smith Building, Cambridge, CB2 0SZ, United Kingdom (second affiliation: National Institute for Health Research Applied Research Collaboration East of England).
3. Praxis Centre for Policy Studies, Tartu mnt 50, Tallinn, 10115, Estonia
4. Medical Research Council Cognition and Brain Sciences Unit, University of Cambridge, 15 Chaucer Road, Cambridge, CB2 7EF, United Kingdom
5. University Counselling Service, University of Cambridge, 2-3 Bene't Place, Lensfield Road, Cambridge, CB2 1EL, United Kingdom (second affiliation: British Association for Counselling & Psychotherapy: Universities and Colleges Division).

***Supplementary Methods***

***Peripheral blood sampling and immunophenotyping.***

Each participant provided a peripheral venous blood sample up to 22.5 mL, drawn between 9 and 10:30 and no fasting was required. Blood samples were collected into S-Monovette® 8.5 mL CPDA_1_ and S-Monovette® 7.5 ml Z-Gel tubes. Z-Gel tubes were centrifuged at 16,000 x g for 15 minutes at room temperature and the serum collected and stored at -70 °C until completion of the study. Serum samples were analysed for cytokine levels using MSD technology in one single batch.

Peripheral blood mononuclear cells (PBMCs) collected in the CPDA_1_ tube were isolated by centrifugation over Histopaque 1077 (Sigma) at 700 x g for 20 minutes at room temperature. PBMCs at the interface were collected, rinsed twice with rinsing buffer (1 x PBS, 2 mM EDTA), and suspended in running buffer (1xPBS, 2mM EDTA, 0.5% BSA). All samples were processed within 2 hours of venepuncture.

Each of 5 different antibody cocktails (**Table S1**) were used to label 10^6^ PBMCs along with 2 μl Fc block (Miltenyi Biotec) and 0.75 μl LIVEDEAD Zombie Aqua™ Fixable Viability Dye (Biolegend®) by incubation in a final volume of 100 μl for 20 minutes at 4°C in the dark. All antibodies details are listed in **Table S2**. After incubation, samples were rinsed with running buffer then resuspended in FACS fix (1% formaldehyde, 111mM glucose and 0.02% sodium azide in PBS). They were stored at 4°C overnight and then acquired using a 4-laser BD Fortessa flow cytometer. Single colour compensation tubes (BD CompBeads) were prepared for each of the fluorophores used and acquired at the start of each flow cytometer run.

Samples were gated in FlowJo v10.2 according to the schema set out in **Figure S1**. The number of cells falling within each gate was recorded. For analysis, these were expressed either in relative terms, as a proportion of a ‘parent’ population (**Table S3**).

Cytometry data from 2 participants did not pass quality control criteria and therefore these subjects were excluded from further analysis.

**Table S1. Antibody staining combinations used for flow cytometry (FACS) analysis.** Aliquots of PBMCs were stained with one of five antibody combinations prior to FACS analysis. The laser and band pass filter combination used to detect each antibody is also shown.

|  | | Antibody combinations | |  |  |  |
| --- | --- | --- | --- | --- | --- | --- |
| Laser | Filter | 1 | 2 | 3 | 4 | 5 |
| 488nm | **530/30** | CXCR3 | CXCR5 | IgD | CD56 | CXCR3 |
|  | **695/40** | CD45RA | CD45RA | CD24 | CD123 | CD45RA |
| 561nm | **582/15** | CCR7 | CD25 | IgG | CD116 | CXCR5 |
|  | **610/20** | CD38 |  |  |  |  |
|  | **780/60** | CCR6 | CCR4 | CD27 | CD11c | CCR6 |
| 640nm | **670/14** | CD161 | CD127 | CD38 | CD16 | PD1 |
|  | **780/60** | CD4 | CD4 | CD20 | CD19/CD20 | CD62L |
| 405nm | **450/50** | HLA-DR | HLA-DR | CD19 | HLA-DR | CD4 |
|  | **525/50** | Live-dead | Live-dead | Live-dead | Live-dead | Live-dead |
|  | **605/12** | CD3 | CD3 | CD3 | CD14 | CD3 |
|  | **655/8** | CD8 |  |  |  | CD8 |

**Table S2. Antibody details.**

| Marker | Fluorochrome | Company | Clone | Catalogue Number |
| --- | --- | --- | --- | --- |
| CXCR3 | AF488 | Biolegend | G025H7 | 353710 |
| CXCR5 | AF488 | Biolegend | J252D4 | 353204 |
| IgD | FITC | BD Biosciences | IA6-2 | 555778 |
| CD56 | FITC | eBioscience | MEM188 | 11-0569-42 |
| CD45RA | PerCP-Cy5.5 | eBioscience | HI100 | 45-0458-42 |
| CD24 | PerCP-Cy5.5 | BD Biosciences | ML5 | 561647 |
| CD123 | PerCP-Cy5.5 | eBioscience | 6H6 | 45-1239-42 |
| CCR7 (CD197) | PE | Biolegend | G043H7 | 353204 |
| CD25 | PE | eBioscience | BC96 | 12-0259-42 |
| IgG | PE | BD Biosciences | G18-145 | 555787 |
| CXCR5 | PE | eBioscience | MU5UBEE | 12-9185-41 |
| CD38 | PE-dazzle | Biolegend | HIT2 | 303538 |
| CCR6 (CD196) | PECy7 | BioLegend | GO34E3 | 343418 |
| CCR4 (CD194) | PEVio770 | Miltenyi | REA279 | 130-103-814 |
| CD27 | PECy7 | eBioscience | 323 | 25-0279-42 |
| CD11c | PEVio770 | Miltenyi | MJ4-27G12 | 130-099-712 |
| CD38 | APC | Biolegend | HB-7 | 356606 |
| CD127 (IL-7R) | AF647 | eBioscience | eBioRDR5 | 17-1278-42 |
| CD16 | APC | eBioscience | eBioCD16 | 17-0168-42 |
| CD161 | APC | eBioscience | HP-3910 | 17-1619-42 |
| PD1 (CD279) | APC | eBioscience | J105 | 17-2799-42 |
| CD4 | APC-eF780 | eBioscience | RPA-T4 | 47-0049-42 |
| CD20 | APC-eF780 | eBioscience | 2H7 | 47-0209-42 |
| CD19 | APC-eF780 | eBioscience | HIB19 | 47-0199-42 |
| CD62L | APC-eF780 | eBioscience | DREG-56 | 47-0629-42 |
| HLA-DR | eFluor 450 | eBioscience | L243 | 48-9952-42 |
| CD19 | eFluor 450 | BD Biosciences | HIB19 | 560353 |
| CD4 | eFluor450 | eBioscience | OKT4 | 48-0048-42 |
| CD3 | eVolve605 | eBioscience | OKT3 | 83-0037-42 |
| CD14 | eVolve605 | Biolegend | M5E2 | 301834 |
| CD3 | eVolve 655 | eBioscience | OKT3 | 86-0037-42 |
| CD8 | eVolve 655 | eBioscience | RPA-T8 | 86-0088-42 |
| Live-Dead | Aqua | Biolegend | | 423102 |

**Table S3. Definition of immune cell populations used for analysis.**

| Population Name | Lineage markers |
| --- | --- |
| B cells | CD3^-^ CD19^+^ |
| CD4+ T helper cells (Th) | CD3^+^ CD4^+^ |
| CD8+ Cytotoxic T cells (Tc) | CD3^+^ CD8^+^ |
| Monocytes | CD3^-^ CD19^-^ CD20^-^ CD56^-^ CD14^+^ |
| Natural killer (NK) cells | CD3^-^ CD19^-^ CD20^-^ CD14^-^ CD56^+^ |

**
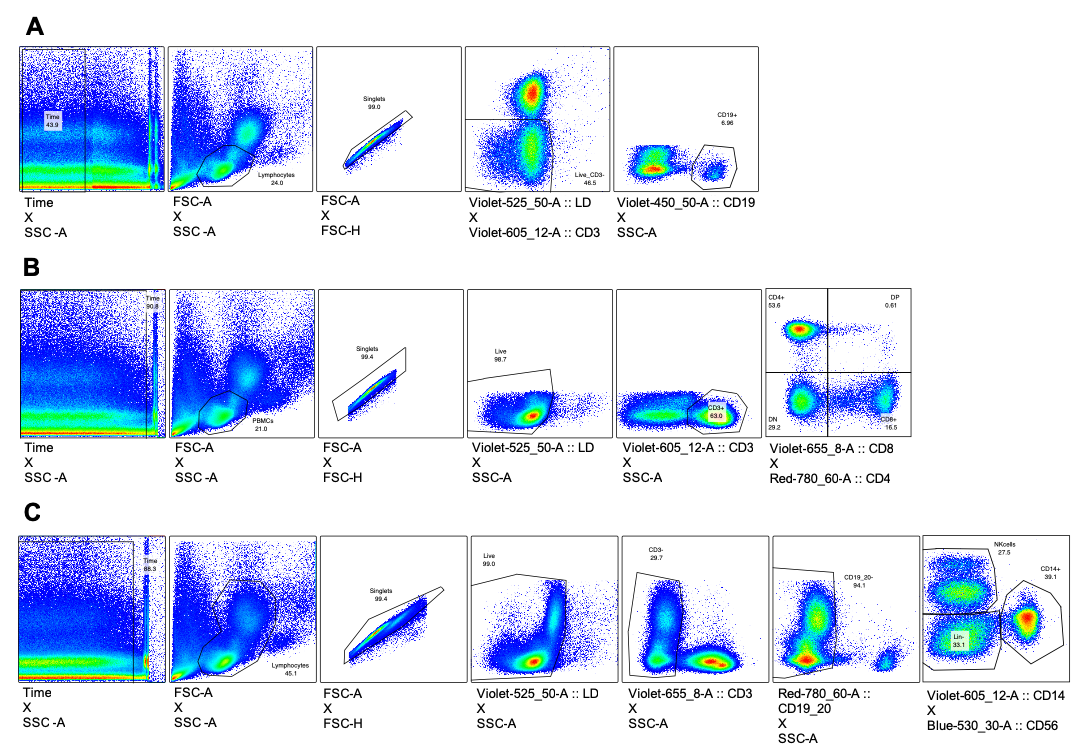
**

***Figure S1: Flow cytometry gating strategy for the immune cell populations used for analysis.***

PBMCs were stained with one of five antibody staining combinations (see **Table S2**) and analysed by flow cytometry. Panels show the progressive gating strategies to obtain B-cell subsets (A), CD4^+^ and CD8^+^ T-cell subsets (B) and CD14^+^ and NK cell subsets (C).


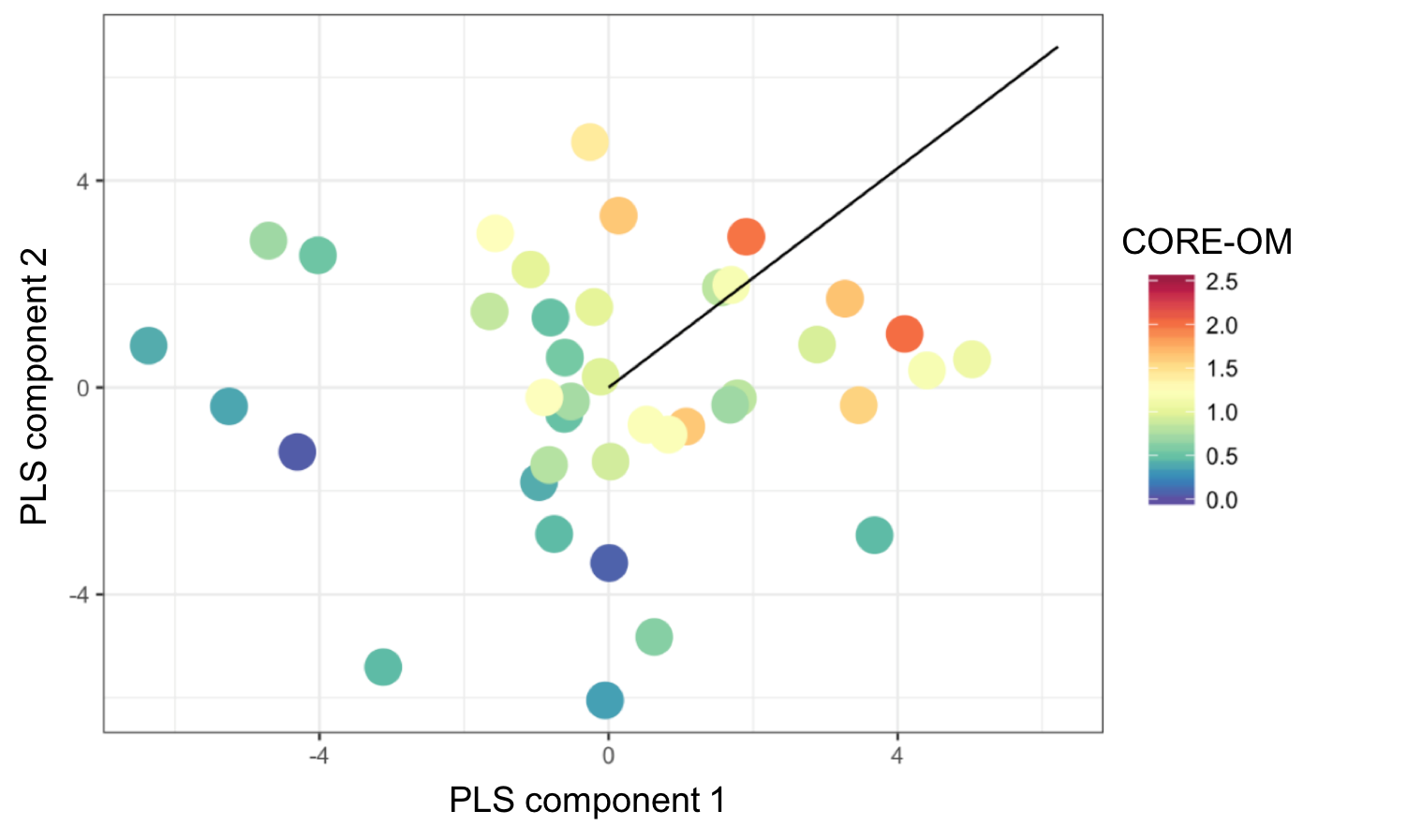


***Figure S2. Participant scores on the first two PLS components, coloured by CORE-OM score.***

*The black line indicates the direction of the response variable (CORE-OM). Each circle represents participant scores on the first two PLS components.*

P = 0.738 ns


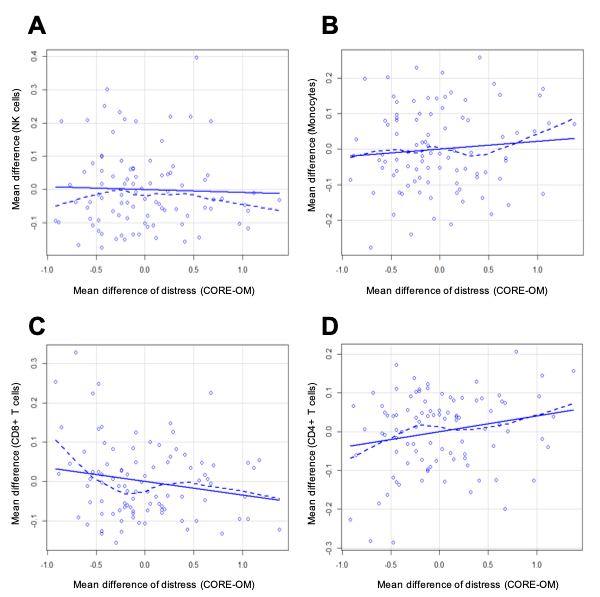


***Figure S3. Relationship between mean differences of CORE-OM and mean differences of immune cell counts.*** To obtain the mean differences, the distances of each CORE and immune cells subset data points from the mean CORE and the subset at their respective time points were calculated. Shown are NK cells (A), Monocytes (B), CD8+ T cells (C) and CD4+ T cells (D).


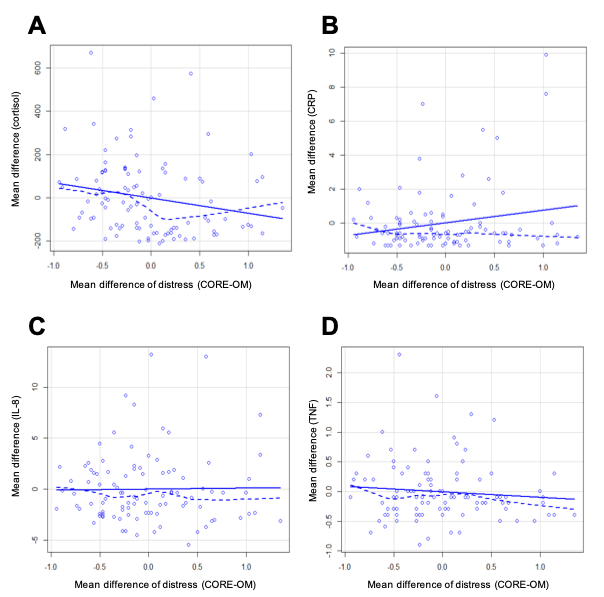


***Figure S4. Relationship between mean differences of CORE-OM and mean differences of humoral markers.*** To obtain the mean differences, the distances of each CORE and humoral marker data points from the mean CORE and the humoral marker at their respective time points were calculated. Shown are cortisol (A), CRP (B), IL-8 (C) and TNF-α (D).
